# Undiagnosed connective tissue diseases: High prevalence in pulmonary arterial hypertension patients

**DOI:** 10.1097/MD.0000000000004827

**Published:** 2016-09-30

**Authors:** Lorenzo Cavagna, Veronica Codullo, Stefano Ghio, Carlo Alberto Scirè, Eleonora Guzzafame, Laura Scelsi, Silvia Rossi, Carlomaurizio Montecucco, Roberto Caporali

**Affiliations:** a Division of Rheumatology; b Department of Cardiology, University and IRCCS Foundation Policlinico S. Matteo, Pavia; c Epidemiology Unit, Italian Society for Rheumatology, Milan, Italy.

**Keywords:** antinuclear antibodies, connective tissue diseases, pulmonary hypertension, Raynaud phenomenon

## Abstract

Among different subgroups of pulmonary arterial hypertension (PAH), those associated with connective tissue diseases (CTDs) have distinct hemodynamic and prognostic features; a correct etiologic diagnosis is thus mandatory.

To estimate frequency and prognosis of previously undiagnosed CTDs in a suspect idiopathic (i) PAH cohort.

Consecutive patients with PAH confirmed by right heart catheterization referred at the Cardiology Division of our Hospital without a previous rheumatological assessment or the occurrence of other conditions explaining PAH were checked for CTD by a clinical, laboratory, and instrumental evaluation. Survival in each group has also been analyzed.

In our study 17 of 49 patients were classified as CTD-PAH, corresponding to a prevalence (95% CI) of 34.7% (21.7–49.6%). ANA positivity had 94% (71.3–99.9%) sensitivity and 78.1% (60–90.7%) specificity for a diagnosis of CTD-PAH; Raynaud phenomenon (RP) showed 83.3% (51.6–97.9%) sensitivity and 100% (90.5–100%) specificity for the diagnosis of Systemic Sclerosis (SSc)-PAH. At diagnosis, SSc patients were older and had a lower creatinine clearance compared with iPAH and other CTD-PAH. After a median follow-up of 44 (2–132) months, 18 of 49 (36.7%) patients died: 31.2% in the iPAH group, 20% in the CTD-, and 58.3% in the SSc-PAH group. Mortality was significantly higher in SSc-PAH (HR 3.32, 1.11–9.95, *P* <0.05) versus iPAH.

We show a high prevalence of undiagnosed CTDs in patients with iPAH without a previous rheumatological assessment. All patients with RP were diagnosed with SSc. Our data stress the importance of a rheumatological assessment in PAH, especially because of the unfavorable prognostic impact of an associated SSc.

## 1 Introduction

Pulmonary arterial hypertension (PAH) is a pathophysiologic alteration associated with a wide range of conditions,^[1]^ including connective tissue diseases (CTDs). PAH prevalence in CTDs varies greatly, with the highest rates reported in mixed connective tissue disease (MCTD) (21–29%), systemic lupus erythematosus (SLE) (up to 14%),^[2]^ and systemic sclerosis (SSc) (nearly 10%).^[3]^ PAH is more rarely observed in inflammatory myopathies^[4]^ and Sjögren syndrome (SS).^[2]^ CTD-associated PAH has a poorer prognosis^[5,6]^ and worse cardiac dysfunction compared with idiopathic PAH (iPAH).^[7]^ Immunosuppressant drugs may be helpful in some CTDs,^[8,9]^ thus enlarging the spectrum of available therapies.

Challenges of PAH diagnosis include clinically undetected CTDs that may not have overtly developed or have been left unidentified until PAH clinical manifestations, analogously to what we have shown in other conditions such as pregnancy.^[10]^ International guidelines for PH diagnosis and management^[11]^ recommend identifying associated CTD and recently several new diagnostic/classification criteria have been proposed for many CTDs.^[12–14]^. Starting from these assumptions, we aimed to evaluate the prevalence of undiagnosed CTDs among patients referred to a tertiary cardiology center to perform right heart catheterization (RHC), without a previous rheumatology referral.

## 2 Methods

This study was approved by the local Ethic Committee of the IRCCS Policlinico San Matteo Foundation. After ethical approval and informed consent obtained, the first 50 patients with RHC-confirmed precapillary PAH, consecutively assessed at the Cardiology Division of the University Hospital of Pavia from September 2008 to November 2011, and satisfying inclusion criteria were included in the analysis. Inclusion criteria were: no previous CTDs diagnosis, no previous rheumatology assessments, no overt or suspect concomitant diseases or conditions explaining PAH before the referral to the tertiary cardiology center. Patients have been referred to the cardiologist in the suspicion of PAH by their general practitioner or by a nonrheumatologist specialty doctor who evaluated them in the Accidents and Emergencies Department with symptoms of right heart failure.

Patients were evaluated for CTDs symptoms/signs occurrence by two expert rheumatologists (LC, and SR) and underwent nailfold-capillaroscopy, Schirmer test, and unstimulated whole saliva flow. Chest computed tomography for both interstitial lung disease (ILD) evaluation (high-resolution) and thromboembolisms exclusion (angiographic technique) was carried out. Laboratory tests included plasma creatinine, brain natriuretic peptide (BNP), ANA (indirect immunofluorescence), anti-ENA (ELiA), anti-dsDNA (indirect immunofluorescence), anti-cardiolipin, anti-B2glicoprotein I (ELISA) antibodies, Lupus Anticoagulant (LAC), C3, C4, and direct Coombs test. Autoantibodies and LAC were confirmed in 2 determinations 6 weeks apart. Creatinine clearance was calculated using Mayo quadratic formula.^[15]^ Well-established and recently published classification criteria were used for the identification of CTD patients.^[12–14,16–20]^ NYHA class (due to the limited number of patients, classes I and II were considered one group as were classes III and IV) and RHC results were collected and compared. Survival for both incident and prevalent patients was computed from the time of the first RHC.

### 2.1 Statistics

Prevalence of undiagnosed CTDs in iPAH patients was calculated and presented with a 95% exact confidence interval (95% CI). Sensitivity and specificity of ANA and Raynaud phenomenon for the diagnosis of CTD were calculated. The groups’ categorical variables (sex, NYHA-class) were compared using the Fisher exact test. Continuous variables (age, BNP, creatinine clearance, mean PAPs, cardiac index, pulmonary vascular resistance at first RHC) were compared using nonparametric tests. Overall differences were tested using the Kruskal–Wallis equality-of-populations rank test. Post hoc paired comparisons were made with iPAH as the reference category using the Mann–Whitney two-sample statistic. Linear correlations between continuous variables were evaluated calculating the Spearman rank correlation coefficient. The log-rank test and Cox regression analyses were used to analyze survival. A 2-sided *P* value <0.05 was considered statistically significant. All analyses were performed using Stata 11.1 (Stata Corp, College Station, TX).

## 3 Results

Of the first 50 patients referred to the rheumatological assessment from the cardiologists, one denied consent to the study. Finally, we analyzed 49 patients (36 females, 13 males). After the rheumatological (re)-assessment, 32 patients (65%; 21 F, 11 M) were confirmed suffering of an idiopathic form of PAH, whereas 17 (15 F, 2 M) were classified as having a CTD-associated condition, corresponding to an estimated prevalence (95%CI) of 34.7% (21.7–49.6%). In particular, 12 had SSc (25%, 6 limited cutaneous, 4 limited, 2 patients satisfied only the 2013 ACR/EULAR Classification criteria; 11 F, 1 M), 2 SLE (4%, 2 F), 2 UCTD (4%, 1 F, 1 M), and 1 SS (2%, 1 F). The two SSc patients satisfying the 2013 ACR/EULAR criteria were first classified as UCTD at the beginning of the study.

CTDs features observed in reclassified patients were mainly represented by Raynaud phenomenon (10 SSc), dysphagia (7 SSc), teleangectasias (6 SSc), and sclerodactyly (6 SSc). The 2 SSc patients without Raynaud phenomenon were ANA positive (homogeneous pattern) and had puffy fingers, pitting scars, teleangectasias, nailfold capillaroscopy alterations, thus satisfying the 2013 ACR/EULAR classification criteria^[14]^ independently of PAH inclusion. One patient with UCTD presented arthritis, fever, mild ILD and ANA positivity, the other one was characterized by photosensitivity, alopecia, ANA test, dry eye with positivity of Schirmer test. One SLE patient had arthritis, oral ulcers, leukopenia, thrombocytopenia, ANA test positive, and hypocomplementemia, the other one photosensitivity, malar rash, oral ulcers, serositis, ANA, and antiphosholipid antibodies. Sjogren syndrome was diagnosed according to symptoms (dry eyes and mouth), positivity of anti-Ro antibodies, Schirmer test, unstimulated whole saliva flow, and minor salivary gland biopsy. The main laboratory and instrumental tests observed in reclassified patients were ANA positivity (titers ≥1:160) (11 SSc, 5 other CTDs), anticentromere antibody positivity (9 SSc), and positive nailfold-capillaroscopy (11 SSc). Several CTD features were also observed in iPAH patients (ANA in 8 cases, antiphospholipid-antibodies/LAC and alopecia in 2, fever and anti-Ro positivity in 1 respectively) but did not affect patients’ reclassification. ANA positivity was associated with a sensitivity of 94% (95% CI 71.3–99.9%) and a specificity of 78.1% (60–90.7%) for a diagnosis of CTD-associated-PAH; while Raynaud phenomenon was associated with a sensitivity of 83.3% (95% CI 51.6–97.9%) and a specificity of 100% (90.5–100%) for a diagnosis of SSc-associated-PAH. Table 1 gives all CTD characteristics observed in our study sample according to patients’ reclassification.

**Table 1:**
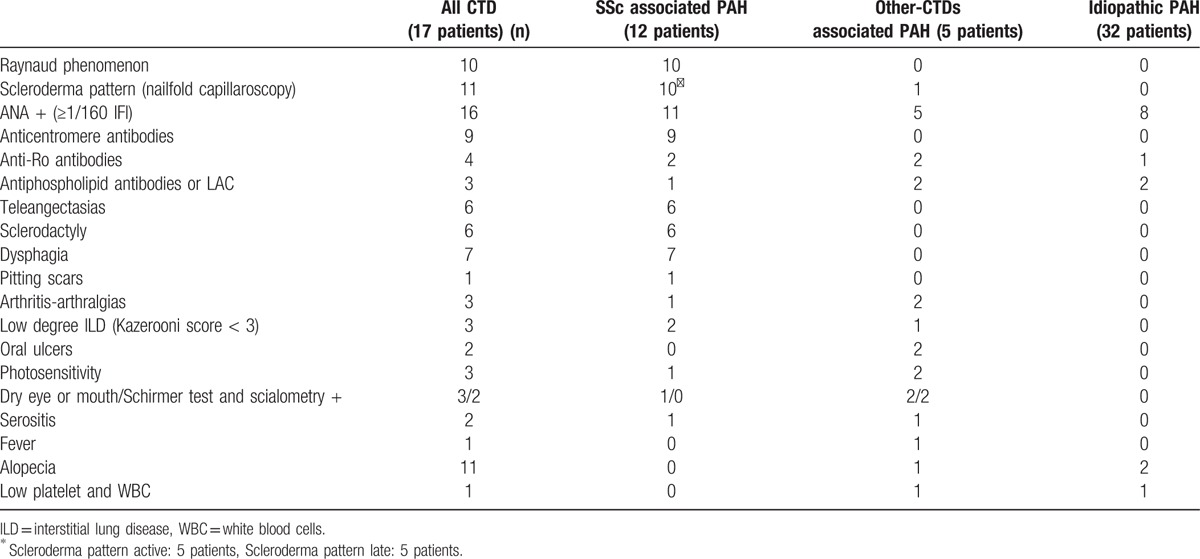
Connective tissue disease (CTD) signs and symptoms observed in our study population of PAH patients, according to their final diagnosis.

At PAH diagnosis, SSc patients were older than those with iPAH (73 (64.5–76.5) vs 58 (46.5–73) years, *P* = 0.01), while the age of iPAH vs all-CTDs patients was not statistically different (*P* = 0.24). There was no difference in sex prevalence between the various groups. The delay between respiratory symptoms onset and the first RHC did not differ between CTDs and iPAH as well as the distribution of baseline NYHA classes (see Table 2). BNP levels were lower in other-CTDs (215 (73–471) pg/mL) and iPAH (median 214 (104–432) pg/mL) than in SSc (267.5 (202.5–667)) but the difference did not reach statistical significance. Creatinine clearance was reduced in SSc (74.9 (59.6–86.1) mL/min/1.73 m^2^) with respect to iPAH (93.6 (62.8–106.8) mL/min/1.73 m^2^, *P* = 0.03). For what concerns the hemodynamic features, mean basal PAPs, cardiac index (CI), and pulmonary vascular resistances (PVR) did not vary between groups. Table 2 provides the demographic, clinical, laboratory, and RHC features in all groups.

**Table 2:**
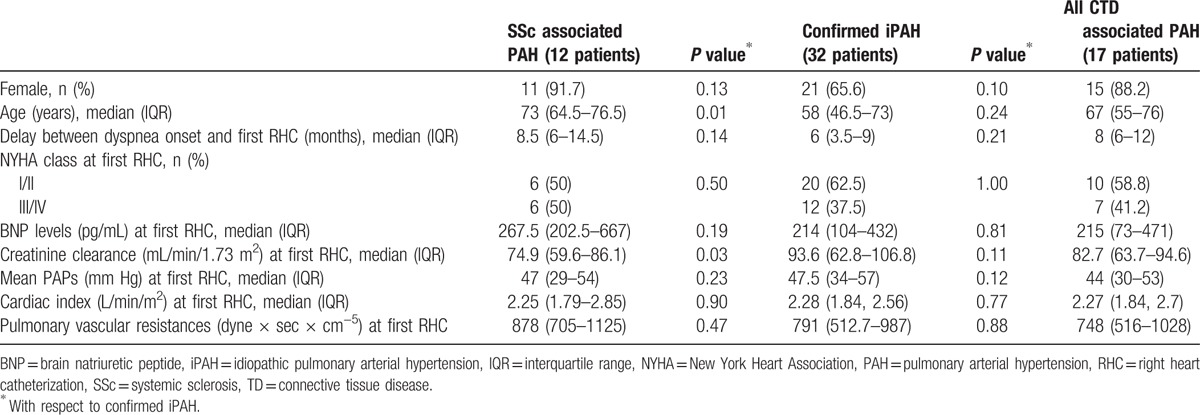
Main demographic, clinical, laboratory, and RHC differences between groups.

Finally, we observed an inverse linear relationship between mPAP and CI (rho −0.62, *P* = 0.007) in all CTD patients while this association was not significant in patients with iPAH (rho −0.11; *P* = 0.54).

**Figure 1:**
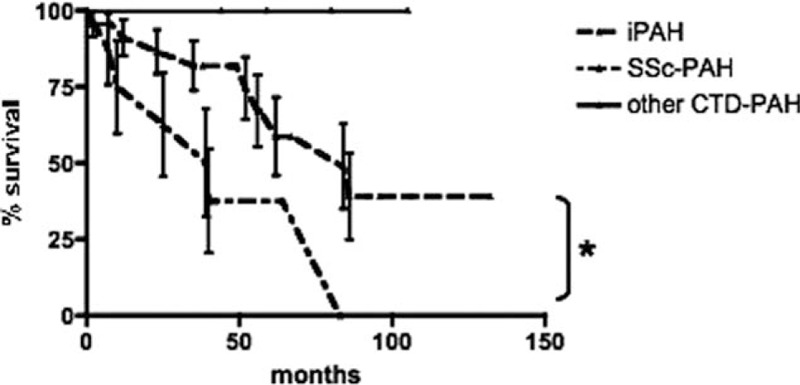
Kaplan–Meier survival curves of the different PAH diagnostic groups. ^∗^*P* <0.05.

After a median follow-up of 44 (2–132) months, 18 of 49 (36.7%) patients were deceased: 10 of 32 (31.2%) in the iPAH group, 1 of 5 (20%) in the CTD, and 7 of 12 (58.3%) in the SSc-PAH group. Survival was significantly lower in SSc-PAH (HR 3.32, 95% CI 1.11–9.95, *P* < 0.05) versus iPAH while there was no statistically significant difference between non-SSc CTDs and iPAH (Fig. 1). SSc patients were older than iPAH and correcting the effect of an SSc diagnosis with age with respect to mortality in an adjusted analysis resulted in a yet positive but no longer significant association compared with crude analysis (HR 1.61, 0.54–4.83).

## 4 Discussion

In our study, more than 30% of patients suspected or diagnosed with iPAH had an undiagnosed CTDs. Even if all CTDs should be potentially considered, SSc is the most frequent one (24.4%). All patients with this disease are now routinely screened for PAH occurrence.^[21,22]^ The features of PAH in established SSc have been described in terms of epidemiology^[3,23]^ and unique features with respect to iPAH.^[5–7,24]^ Tools such as the Cochin risk prediction score^[25]^ or the DETECT score^[26]^ could help clinicians to select SSc patients at higher risk for PAH development. International guidelines (Authors/Task Force, Galie et al 2015^[11]^) suggested that occasional patients diagnosed with iPAH could have an associated CTD, thus justifying the screening for CTDs in iPAH patients;^[11]^ however, to date, no studies evaluating this aspect are available.

Classification criteria for CTDs are continuously being updated by international study groups to improve their sensitivity and specificity, especially in the early phases of diseases.^[12–14]^ In the case of SSc, new criteria have been released in 2013 and PAH is one of the new items included, acknowledging the fact that this manifestation could represent the onset of the disease as the first non-Raynaud symptom.^[14]^ In SSc duration of Raynaud phenomenon can span many years before the disease manifests itself.^[27]^ The most important result of our study is not only that undiagnosed CTD may be frequently identified in PAH patients, but also that Raynaud phenomenon is strictly associated with a subsequent diagnosis of SSc. The reported prevalence of RP in iPAH in the literature ranges from 5% to 30%,^[28–31]^ and this might be accounted by the high prevalence of an undiagnosed rheumatic disease in these cohorts. Our results clearly indicate that a careful rheumatological evaluation (including nailfold capillaroscopy) should be considered to increase diagnosis specificity in these patients and to reduce the risk of a hidden SSc spectrum disorder. Beside capillaroscopy, autoantibody profiling is also mandatory for the screening of SSc and other CTDs in general.

We have previously demonstrated that a simple questionnaire could identify a population with a 30% frequency of undiagnosed CTD in otherwise healthy pregnant women.^[10]^ Results from the present study suggest the potential utility of a similar approach in PAH patients. Analogously to the effort made in the field of interstitial lung disease with the recent introduction of the concept of interstitial pneumonia with autoimmune features^[32]^ we propose that a population of PAH patients show autoimmune features which may rely undetected at a first examination and which require a multidisciplinary expertise and the mandatory performance of ANA testing and capillaroscopy. Furthermore, this approach is essential considering the continuous progresses in the classification and diagnosis of rheumatic conditions.

Moreover, the identification of an underlying CTD is crucial as the disease course is frequently complicated by disease-related manifestations other than PAH which may affect the prognosis of these patients. In SSc, for example, ILD may develop at any time during the course of the disease and this may complicate PH classification (group 1 PAH vs group 3 PH associated with chronic lung diseases) and subsequent treatment.^[33]^ CTD-associated PAH may benefit from immunosuppressant therapies beside classical therapies: SLE or MCTD patients may respond to treatments combining cyclophosphamide and glucocorticoids,^[8]^ particularly in the early phases of the disease. To date, no cases of UCTD-associated PAH have been reported in the literature and the present study is the first to link such condition to PAH. Although age may discriminate patients with SSc from those with iPAH,^[7]^ our results showed that this difference is not helpful in case of other CTDs occurrence. As previously described,^[34]^ renal function was lower in SSc patients and this is not unexpected given the frequent subclinical kidney involvement in the disease.^[35]^ Given the impact of renal dysfunction on PAH prognosis,^[36]^ this may partially explain the worse survival of SSc-associated PAH than iPAH.^[7]^ Demographics, clinical, laboratory and RHC results between groups were compared, in order to identify factors that may help clinicians in CTD identification and to assess hemodynamic differences between these patients. RHC parameters were substantially similar in the head-to-head analysis. In CTDs we observed an inverse linear relationship between mPAP and CI that was lacking in iPAH, thus suggesting that cardiac function is more compromised in CTD patients. Survival in our cohort is influenced by the diagnosis of SSc in the univariate analysis, but this effect is dependent on the age difference of SSc versus iPAH patients. Anyway, our results are in line with the literature suggesting SSc-PAH as a definite and worse—in prognosis—subset of patients. Despite the availability of new promising therapies the survival of patients with SSc, especially when it is complicated with pulmonary hypertension, is still significantly reduced when compared with the general population.^[37]^ Therefore, early diagnosis of patients with this condition is of major importance. Assessment of patients with Raynaud phenomenon to disclose patients with connective tissue diseases may be a key symptom to disclose CTDs and SSc in particular.

The small cohort that we analyzed is responsible for the lack of significance in the multivariate analysis of survival because of the paucity of events in our follow-up. Beyond this latter limit, we also acknowledge that we performed a single-center study that is therefore open to the risk of selection bias and also to criticisms on the feasibility of the application of such a quick referral where the cardiologist and the rheumatologist are not in close cooperation or even not in the same hospital. In this view, we must consider that one recommendation of the management of PH is to refer patients to a tertiary center, which in our view has to encompass a rheumatology clinic.

## 5 Conclusions

Our results show a high prevalence of undiagnosed CTDs in patients classified or suspected with iPAH and without previous rheumatology referrals. SSc is the preeminent CTD diagnosed in the setting and Raynaud phenomenon is the main suspect finding of SSc that all clinicians should assess. We highlight the importance of a multidisciplinary approach, which may result helpful with regards to classification and follow-up/therapeutic implications.
